# The influence of natural and anthropic environmental variables on the structure and spatial distribution along longitudinal gradient of macroinvertebrate communities in southern Brazilian streams

**DOI:** 10.1093/jis/14.1.13

**Published:** 2014-01-01

**Authors:** Andrea Vanessa Batalla Salvarrey, Carla Bender Kotzian, Márcia Regina Spies, Bruna Braun

**Affiliations:** 1 Programa de Pós-Graduação em Biodiversidade Animal, Centro de Ciências Naturais e Exatas, Universidade Federal de Santa Maria. Faixa de Camobi km 9, 97105-900, Santa Maria, RS, Brazil; 2 Departamento de Biologia e PPG Biodiversidade Animal, Centro de Ciências Naturais e Exatas, Universidade Federal de Santa Maria. Faixa de Camobi km 9, 97105-900, Santa Maria, RS, Brazil; 3 Universidade Federal do Pampa, Av. Antônio Trilha, 1847, 97300-000, São Gabriel – RS, Brazil; 4 Curso de Graduação em Ciências Biológicas, Centro de Ciências Naturais e Exatas, Universidade Federal de Santa Maria. Faixa de Camobi km 9, 97105-900, Santa Maria, RS, Brazil

**Keywords:** aquatic insects, landscape, multiple scales, Neotropical region, river order

## Abstract

Southern Brazilian rivers and streams have been intensively affected by human activities, especially agriculture and the release of untreated domestic sewage. However, data about the aquatic macroinvertebrates in these streams are scarce and limited to only certain groups. In addition, studies focusing on the structure and spatial distribution of these communities are lacking. This study analyzed the effects of natural and anthropic variables on the community structure of macroinvertebrates along a longitudinal gradient in three microbasins located in a region of landscape transition in the state of Rio Grande do Sul, Brazil. Sampling was conducted in the Vacacaí-Mirim River (August 2008) and in the Ibicuí-Mirim and Tororaipí rivers (August 2009) following an environmental gradient including 1 ^st^ , 2 ^nd^ , 3 ^rd^ , and 4 ^th^ order segments. Local natural factors that were analyzed include water temperature, pH, electrical conductivity, dissolved oxygen, substrate granulometry, and the presence of aquatic vegetation. Anthropic variables that were analyzed include including bank erosion, land use, urbanization, riparian deforestation, and fine sediments input. A total of 42 families and 129 taxa were found, with predominance of environmentally tolerant taxa. Geological context (landscape transition and large hydrographic basins) tended to influence natural environmental factors along the rivers’ longitudinal gradients. However, changes in anthropic variables were not affected by these geological differences and therefore did not correlate with patterns of spatial distribution in macroinvertebrate communities. Only 1 ^st^ order stream segments showed a community composition with high richness of taxa intolerant to anthropic disturbance. Richness as a whole tended to be higher in 3 ^rd^ to 4 ^th^ order set of segments, but this trend was a result of local anthropic environmental disturbances. Future inventories conducted in similar landscape transition regions of Brazil, for conservation purposes, must consider stream segments of different orders, microbasins, and major basins in order to obtain data that faithfully reflect the regional diversity. Additionally, it is necessary to consider environmental gradients of land use and anthropic impacts in order to suggest appropriate strategies for conserving the environmental integrity of streams.

## Introduction

Macroinvertebrates play important ecological roles in maintaining the integrity of biological communities in rivers and streams. They are important elements in the trophic chain, constituting prey for vertebrates and participating in nutrient cycling and energy flow ( Cummins 1979 ; Vannote et al. 1980 ). Macroinvertebrate occurrence is intimately related to river environmental characteristics ( Hynes 1970 ; Sullivan et al. 2004 ). Environmental impacts of human activities on rivers and streams affect their macroinvertebrate communities by decreasing richness and altering the composition of stream fauna and flora as a whole (e.g., Rosenberg and Resh 1993 ; Moore and Palmer 2005 ).

The distribution of macroinvertebrate communities in lotic environments is influenced by a wide range of local (altitude, pH, electrical conductivity, substratum, dissolved oxygen, temperature, and aquatic and riparian vegetation), landscape (land use and cover, basin, stream order, surficial geology), and regional (ecoregion) natural environmental factors, including biogeographical factors (see references in Li et al. 2001 ; Feld and Hering 2007 ; Tomanova et al. 2007 ). Anthropic factors, such as deforestation, agriculture, pasture, and urbanization, can also affect macroinvertebrate communities because they alter flow regimes, thermal stability, nutrient release, allochthonous or solar energy flux, and cause soil erosion ( Delong and Brusven 1998 ; Cuffney et al. 2000 ; Miserendino et al. 2011 ). Many environmental variables, including those generated by human activities, are interdependent because rivers are hierarchically structured aquatic systems ( Frissell et al. 1986 ; Hawkins et al. 1993 ), functioning as a ‘nested’ design ( Allen and Starr 1982 ). Riverine faunal assemblages at a particular site can be considered as the product of a series of spatially organized filters, ranging from large (continental, regional, basin, etc.) to small (reach, riffle, stone) scales ( Tonn 1990 ; Poff 1997 ). The River Continuum Concept (Vannote et al. 1980) is the classical means of understanding this process.

Changes in macroinvertebrate communities from upstream to downstream are well known. These changes are related to the influence of altitude on some important natural environmental drivers such as water temperature ( Finn and Poff 2005 , Friberg et al. 2009 ), velocity ( Gagneur 1994 ), and granulometry ( Allan and Castillo 2007 ), which co-varies along the course of a river. Stream order also influences abiotic factors along the longitudinal gradient, such as input of detritus, shading due to the presence of riparian vegetation, and habitat available due to changes in substrate size ( Vannote et al. 1980 ; Rosgen 1994 ). Altitude and stream order also co-vary because they are indissociable. Anthropic factors, although being spatially localized, commonly affect macroinvertebrate communities downstream. Deforestation of riparian vegetation and water pollution due to human activities can alter the structure of these communities by modifying their abundance, composition, and richness ( [Bibr R61][Bibr R77] ).

The annual precipitation of around 1,200 mm in southern Brazil ( Pereira et al. 1989) allows in southern Brazil ( Pereira et al. 1989 ) allows the existence of a rich hydrographic network with numerous perennial streams and rivers. However, historical and geomorphological contexts have led to intense use of the region’s hydrological resources ( Lima et al. 2006 ). In addition, the rapid rate at which lowland rivers and streams have become degraded, especially by damming ( Nunes 2010 ) and deforestation due to agricultural activities ( Rodrigues 2001 ), has not been accompanied by corresponding increases in the knowledge of their faunas, especially aquatic macroinvertebrates. Even simple inventories are scarce, and studies of riverine macroinvertebrate assemblages began only in 2000 ( Stenert et al. 2002 ; Bueno et al. 2003 ). Such studies have become more numerous recently, but many have been conducted at family level ( Milesi et al. 2009 ; Biasi and Restello 2010 ; Hepp et al. 2010 ) or have focused on certain taxa, particularly specific insect orders ( Neri et al. 2005 ; Spies et al. 2006 ; Siegloch et al. 2008 ; Pires et al. 2011 ). Only three studies have investigated macroinvertebrate assemblages as a whole, including taxa identified above the family level ( Piedras et al. 2006 ; Melo 2009 ; Hepp et al. 2013 ).

This combination of rapid environmental degradation of southern Brazilian streams and rivers and the inadequate knowledge of their macroinvertebrate communities calls for an urgent need to understand their diversity and spatial distribution patterns, as well as how natural and anthropic environmental variables influence these patterns. Impacts on the integrity of running waters of the region will not be understood if their inhabitants are not studied.

This study analyzed the composition and spatial distribution of macroinvertebrate communities of three microbasins along a landscape gradient in a region of transitional relief strongly influenced by agriculture and urban land use in extreme southern Brazil. Our hypothesis is that the structure of macroinvertebrate species will be influenced more strongly by environmental variation in different stream orders than by environmental variation in different microbasins. However, this pattern is likely to be impacted by anthropic factors. The influence of local natural and anthropic environmental variables was investigated in order to suggest some requirements for the conservation of macroinvertebrate communities.

## Materials and Methods

### Study area

**Figure 1.**
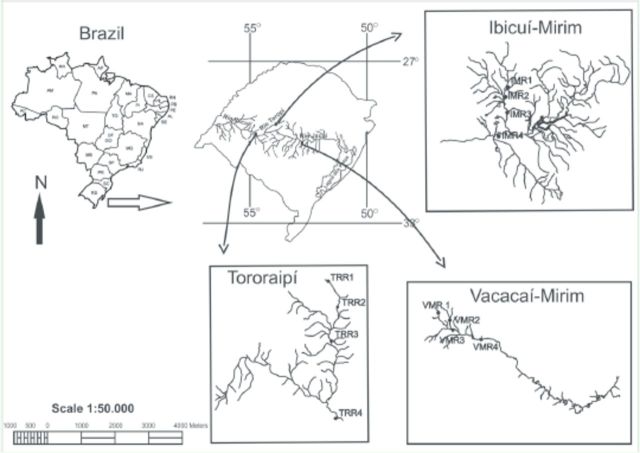
Map showing the locations of the Vacacaí-Mirim, Ibicuí-Mirim, and Tororaipí river microbasins, Rio Grande do Sul, Brazil. High quality figures are available online.

The three studied microbasins are located in the central region of the state of Rio Grande do Sul, Brazil, and are about 30 km from each other ( Figure 1 ). The Vacacaí-Mirim River (VMR) is located in the Municipality of Santa Maria, which has ca. 270,000 inhabitants. The river is 6th order, has a drainage area of ca. 1136 km2 ( Marchesan et al. 2007 ), and belongs to the Jacuí River basin (drainage area of 71,600 km ^2^ ) ( Figure 1 ). The watershed area is primarily given over to farming and ranching ( Cargnin 2010 ). The Ibicuí-Mirim River (IMR) and the Tororaipí River (TRR) belong to the Ibicuí River basin (drainage area of 47,740 km ^2^ ; Paiva et al. 2000 ). The IMR is 5 ^th^ order, drains an area of 33.1 km ^2^ ( Sampaio et al. 2010 ), and is also located in Santa Maria Municipality, but far from the urban area. The TRR is 4 ^th^ order, has a drainage area of 0.15 km ^2^ , and is located in Mata Municipality, which has ca. 6,000 inhabitants. The three microbasins drain agricultural landscapes, however their headwaters suffer less influence from agriculture.

These three microbasins are located in a region of landscape transition, in the foothills of a slope that delimits two geomorphological compartments, the Planalto (uplands) and the Depressão Central (lowlands). The rocks in the slope are formed by the basalts of the Serra Geral Formation, which cover the sandstones of the Botucatu and Caturrita formations ( Robaina et al. 2010 ). These basalts crop out along the slope and also on the riverbanks. In the Planalto, the altitude reaches ca. 500 m a.s.l. Originally, the Planalto and its slope were covered by the floresta estacional semidecídua (semideciduous seasonal forest, a vegetation subtype of the Atlantic Forest) ( *sensu*Prado 2000 ). Currently, only a few fragments of this vegetation can be found, and the riparian vegetation is especially sparse ( Quadros and Pillar 2002 ). In the lowlands of the Depressão Central, the altitude reaches ca. 70 m a.s.l. This region was originally covered by Cerrado (savannah) vegetation ( Quadros and Pillar 2002 ), but at present most of the natural vegetation along streams and rivers has been replaced by rice plantations. The lowland floodplains of the study area are formed by sands deposited by rivers and streams ( Rodrigues and Werlang 2011 ). Some of the headwater springs in these microbasins are located in the foothills of the Planalto slope, and some longer stretches are located in the lowlands of the Depressão Central. The climate of Rio Grande do Sul is “Cfa” according to the Köppen classification, with a mean annual relative humidity of 82% ( Isaía 1992 ), mean annual temperature of 19.2° C, and mean annual precipitation of 1,708 mm (Maluf 2000).

In each microbasin, four sampling sites at least 1 km distance from each other and representing a longitudinal gradient (segments of 1 ^st^ , 2 ^nd^ , 3 ^rd^ , and 4 ^th^ orders) were studied ( Figure 1 ). Sites of the same order of magnitude were chosen in order to show similar environmental conditions ( Table 1 ). Consequently, sites in low-order segments (1 ^st^ and 2 ^nd^ ) were generally located in the foothills of the Planalto slope and had coarser (gravelly) substrates and more well-preserved riparian vegetation than middle-order segments (3 ^rd^ and 4 ^th^ ), which were located in the lowlands of the Depressão Central ( Table 1 ). In the lowlands, the VMR also drains an urban area.

**Table 1.**
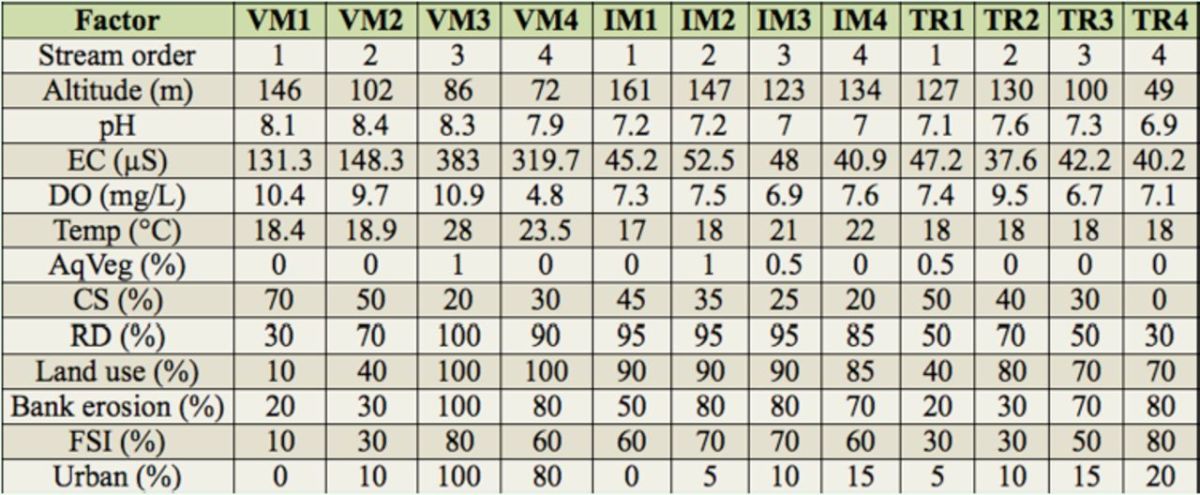
Natural and anthropic environmental characterization of the sampling sites in the Vacacaí-Mirim (VMR, sites 1, 2, 3, and 4) (August 2008), Ibicuí-Mirim (IMR, sites 1, 2, 3, and 4), and Tororaipí (TRR, sites 1, 2, 3, and 4) (August 2009) microbasins, Rio Grande do Sul, Brazil.

EC: electrical conductivity; DO: dissolved oxygen; Temp: temperature; AqVeg: aquatic vegetation; CS: coarse sediments; RD: riparian deforestation; FSI: fine sediments increase; Urban: urbanization

### Sampling

Sampling was conducted in August 2008 (VMR) and August 2009 (IMR and TRR), with a Surber sampler (mesh = 0.25 mm; area = 0.1 m ^2^ ). At each site, three subsamples were taken only once, one near each bank and one in the middle of the streambed. The samples were stored in plastic tubes and fixed with 10% formaldehyde. In the laboratory, they were stained with Rose Bengal, washed in sieves with meshes of 500 and 250 µm, and fixed in 70% ethanol. The specimens were sorted under a stereomicroscope, identified to the lowest taxonomic level possible according to specific references ( Angrisano and Korob 2001 ; Benetti et al. 2006 ; Domínguez et al. 2006 ; Lecci and Froehlich 2007 ; Passos et al 2007 ; Manzo and Archangelsky 2008 ; Domínguez and Fernández 2009 ; Marchese 2009 ), and confirmed with the help of experts (see Acknowledgements).

For each sampling site, the following natural environmental variables were analyzed only once: air temperature (between 09:00 and 16:00), altitude, pH, electrical conductivity (EC), dissolved oxygen (DO), percentage of coarse sediments, and percentage of aquatic vegetation in stream bed. Additionally, the following anthropic environmental variables were analytically estimated in terms of percentage based on local observations and confirmed by the Google Earth ( www.google.com/earth ) satellite images when necessary: bank erosion, % land use, riparian deforestation (RD), fine sediments increase due to bank erosion, and % urbanization (urban).

The monthly means of air temperature and precipitation during the study period and the occurrence of heavy rains prior to sampling were obtained from the Meteorological Station of the Sector of Fitotecnia, Universidade Federal de Santa Maria.

### Data analyses

The values for taxa richness were compared among the three microbasins and among the four segments of different stream orders using the rarefaction method by sample, rescaled by abundance (1,000 permutations; Simberloff 1972 ). The samples of macroinvertebrate communities were organized in two blocks (or treatments) in order to study the two proposed approaches: microbasins (VMR, IMR, and TRR) and stream orders (1 ^st^ , 2 ^nd^ , 3 ^rd^ , and 4 ^th^ ). The comparison of richness was conducted at the highest comparable abundance level among communities (1,700 specimens for microbasin; 2,100 specimens for segments of different orders) ( Gotelli and Entsminger 2001 ). The curves were generated based on 1,000 randomizations by the program EcoSim 700 ( Gotelli and Entsminger 2001 ).

The biotic data matrix used in the statistical analyses was based on the lowest taxonomic unit possible, down to genus level. Taxa with fewer than 10 specimens were disregarded because they introduced a large number of zeros into the analysis, obscuring the patterns and increasing the total inertia of the taxon data ( ter Braak and Šmilauer 2002 ; Titeux et al. 2004 ). The three subsamples (Surber units) obtained in each site were combined in one sample. Additionally, the matrix was Hellinger transformed to reduce double zero influence ( Legendre and Gallagher 2001 ).

The similarity in the structure of the samples of macroinvertebrate communities was evaluated using the chord distance and was posteriorly ordinated by non-metric multidimensional scaling ordination ( Kruskal and Wish 1978 ). The similarity analysis (ANOSIM) of two-way crossed factors (with no replicates) was used to test the difference between two factors: i) samples from segments of different orders, and ii) samples from different microbasins. In the first factor, four levels were included (1 ^st^ , 2 ^nd^ , 3 ^rd^ , and 4 ^th^ order segments), and in the second factor, three levels (the VMR, IMR, and TRR microbasins). The analyses were performed in the program Primer E ( Clarke and Warwick 2001 ).

Redundancy analysis ( van den Wollenberg 1977 ) was used to evaluate the influence of environmental variables on the spatial distribution of the macroinvertebrate communities in the three rivers. This analysis was selected due to the short gradient (SD < 3) shown by the data for macroinvertebrate community composition *(sensu*ter Braak and Šmilauer 2002 ). The gradient length estimates the beta diversity among the communities, i.e., the species turnover. A detrended correspondence analysis was performed to evaluate the length of the gradient (present data: SD = 2.24 on axis I).

In sampling designs showing a spatial structure, a relationship can occur among samples obtained along a geographical space, i.e., spatial autocorrelation ( Legendre and Legendre 1998 ). This relation was evaluated through the construction of a geographical-distance matrix based on the coordinates of the sampling sites. The correlation of the geographical distance matrix with the similarity of the macroinvertebrate community structure was determined by the Mantel test ( Manly 2000 ) using 5,000 permutations generated using the program NTSYSpc 2.10S ( Rohlf 2000 ). In this study, no spatial autocorrelation was detected by the Mantel test (r = 0.14, *p* = 0.14).

In the redundancy analysis, the natural and anthropic environmental variables considered were tested to add in the model through the manual forward stepwise selection procedure ( *p* < 0.05 by the permutation test of Monte Carlo with 999 randomizations). Therefore, only altitude and electrical EC were used in the model to represent the natural variables, and RD, land use, and urbanization degrees were used to represent the anthropic variables. This method was also efficient in removing the multi-colinearity among the explanatory variables, since none of them showed a high inflation factor ( *sensu*ter Braak and Šmilauer 2002 ). Additionally, the Monte Carlo test (999 randomizations) was used to test the significance of the canonical axis and the correlation among taxa and environmental variables ( ter Braak and Šmilauer 2002 ). The biotic matrix data was Helinger-transformed, the environmental percentage variables were arcsine-transformed, and the other environmental variables were square roottransformed and standardized by standard deviation. The transformations were performed to normalize and render the data homoscedastic ( Sokal and Rohlf 1995 ). The environmental data were standardized because of the differences in scale units of the environmental variables (e.g., µS/cm for EC and mg/L for DO) measured ( Clarke and Gorley 2006 ).

The percentage of explicability of each set of environmental variables on the structure of macroinvertebrate communities was assessed through variation partitioning as proposed by Borcard et al. (1992) using redundancy analysis and partial redundancy analysis. In this way, the variation of macroinvertebrate communities was partitioned in four components: i) anthropic environmental variation; ii) natural environmental variation; iii) natural and anthropic intersect variation; and iv) unexplained variation.

## Results

### Environmental variables

Little climatic variation was detected between the two consecutive years of sampling. The mean monthly temperature was 14.3° C in August 2008 and 16.4° C in August 2009. The annual and monthly precipitation in 2008 and 2009 was 1,476 mm and 99.8 mm, and 2,123 mm and 164.5 mm, respectively. No heavy rains were recorded in the week prior to sampling in either year. In general, natural environmental variables also showed little variation among the microbasins and orders studied. However, the VMR presented higher values of pH, EC, and DO and coarser sediments than the IMR and TRR. Small streams exhibited coarser sediments and more well-oxygenated waters than the larger streams and rivers due to their positions in higher altitudes ( Table 1 ). However, anthropic environmental variables tended to vary highly according to stream order in all microbasins ( Table 1 ). With some exceptions (lower RD in 4 ^th^ order segment of the TRR and similar percentages of RD and land use in almost all segments of the IMR), most anthropic variables tended to increase downstream ( Table 1 ).

### Structure of macroinvertebrate communities

In total, 10,985 macroinvertebrates were collected, distributed in 42 families and 129 taxa. The dominant taxa were Simuliidae (14%), Naididae (13%), *Cricotopus* sp. 1 (13%), *Cricotopus* sp. 2 (Chironomidae) (8%), *Paragripopteryx* (Gripopterygidae) (5%), and *Americabaetis* (Baetidae) (5%), which together represented 58% of the total collected. In general, the dominant taxa were the same in all microbasins with differences in dominance rankings. Naididae was more dominant in the VMR, while taxa of Chironomidae were more dominant in the IMR and TRR. The ranking of dominance also varied according to stream order. Naididae was dominant in 4 ^th^ order segments, while some *Cricotopus* taxa dominated 3 ^rd^ order segments, and Simuliidae dominated 2 ^nd^ order segments. In 1 ^st^ order segments, *Cricotopus* taxa and Simuliidae were dominant, but were not as abundant as they were in the larger order segments. Thirty-nine taxa were shared by all three microbasins.Sixty-two were exclusive, occurring only in one microbasin.

**Figure 2.**
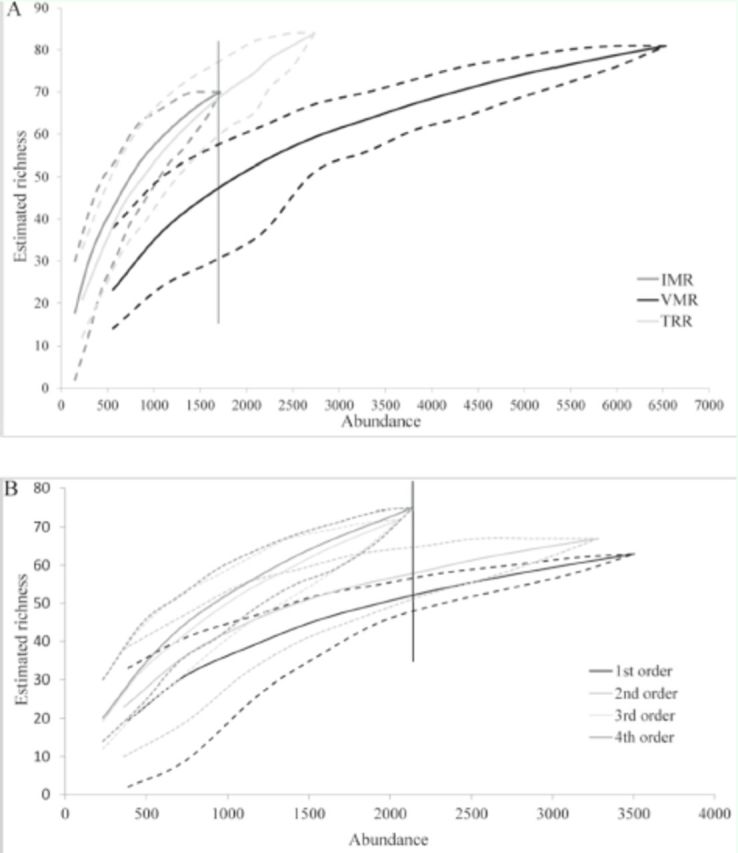
Comparison of estimated richness of benthic macroinvertebrates among (A) microbasins (for a subsample of 1,700 individuals) and (B) different stream order segments (for a subsample of 2,100 individuals) studied in 1st, 2nd, 3rd, and 4 ^th^ order segments of the Vacacaí-Mirim (August 2008), Ibicuí-Mirim, and Tororaipí (August 2009) river microbasins, Rio Grande do Sul, Brazil. The whiskers show the variation around the mean. High quality figures are available online.

No significant differences in richness were recorded among the microbasins at family and lower taxonomic classification levels (TRR: 30 families and 84 lower classifications; VMR and IMR: both 28 families, and 81 and 70 lower classifications respectively). However, the rarefaction test showed higher estimated richness for the TRR and IMR microbasins (there was wide overlapping between confidence intervals), for a comparable sample, i.e., there was higher richness in the TRR and IMR microbasins than the VMR microbasin ( Figure 2A ). The rarefaction method also determined that 3 ^rd^ and 4 ^th^ order segments showed higher richness (25 and 28 families, and 72 and 75 lower taxonomic classifications, respectively) than the 1 ^st^ and 2 ^nd^ order segments (31 and 22 families, and 63 and 67 lower taxonomic classifications, respectively) ( Figure 2B ).

**Figure 3.**
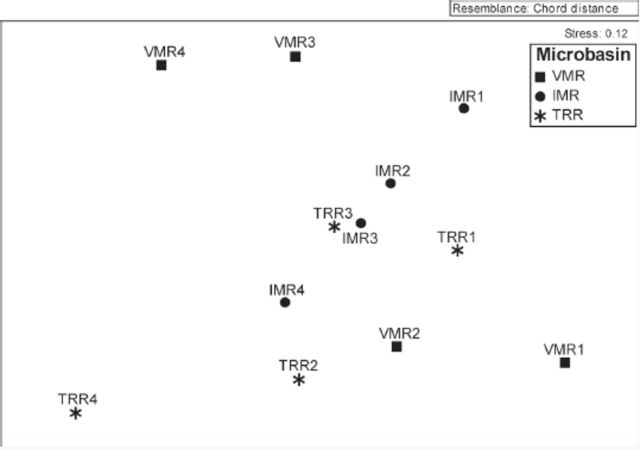
Ordination diagram non-metric multidimensional scaling of the river segments sampled (Vacacaí-Mirim, Ibicuí-Mirim, and Tororaipí) of 1 ^st^ , 2 ^nd^ , 3 ^rd^ , and 4 ^th^ orders and B) of the microbasins sampled (VMR = Vacacaí-Mirim, IMR = Ibicuí-Mirim, and TRR = Tororaipí), in August 2008 (VMR) and August 2009 (IMR, TRR), Rio Grande do Sul, Brazil. High quality figures are available online.

The non-metric multidimensional scaling ordination depicted the same result as the rarefaction method, confirming a trend toward segregation among macroinvertebrate communities of the low orders from the higher ones ( Figure 3 ). The ANOSIM test presented no significant result for both approaches (microbasins: R = 0, *p* = 0.42; order: R = 0.295, *p* = 0.14).

### Influence of natural and anthropic environmental variables on macroinvertebrate communities

**Figure 4.**
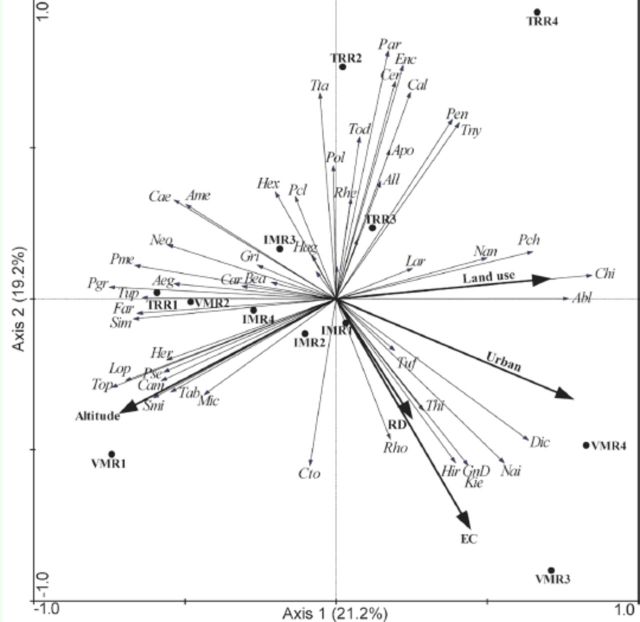
Ordination diagram of samples and taxa within macroinvertebrate communities used in redundancy analysis constrained by natural and anthropic environmental variables of Vacacaí-Mirim River (VMR), August 2008, and Ibicuí-Mirim (IMR) and Tororaipí (TRR) rivers, August 2009, Rio Grande do Sul, Brazil. Nem: Nematoda, All: Alluroididae, Enc: Enchytraeidae, Nai: Naididae, Tuf: Tubificidae, Hir: Hirudinea, Ame: *Americabaetis,* Apo: *Apobaetis,* Cam: *Camelobaetidius,* Par: *Paracloeodes,* Far: *Farrodes,* Hag: *Hagenulopsis,* Tod: *Tricorythodes,* Top: *Tricorythopsis,* Cae: *Caenis,* Gri: *Gripopteryx,* Pgr: *Paragripopteryx,* Tup: *Tupiperla,* Pro: *Progomphus,* Her: *Heterelmis,* Mic: *Microcylloepus,* Neo: *Neoelmis,* Hex: *Hexacylloepus,* Pse: *Psephenidae,* Sim: Simullidae, Cer: Ceratopogonidae, Bea: *Beardius,* Cal: *Caladomyia,* Chi: *Chironomus,* Dic: *Dicrotendipes,* Kie: *Kiefferulus,* Pch: *Parachironomus,* Pol: *Polypedilum,* Rhe: *Rhe-otanytarsus,* , Abl: *Ablabesmyia,* Lar: *Larsia,* Pen: *Pentaneura,* Tny: *Tanypus,* Car: *Cardiocladius,* Cry: *Corynoneura,* Cto: *Cricotopus,* Lop: *Lopescladius,* Nan: *Nanocladius,* Pcl: *Paracladius,* Pme: *Parameotricnemus,* Rho: *Rheocricotopus,* Thi: *Thienemanniella,* GnD: Gen et sp. indet. D, Tab: Tabanidae, Smi: *Smicridea,* Aeg: *Aegla.* High quality figures are available online.

The redundancy analysis axes were significantly different (F = 2.17, *p* < 0.001). The first two axes together summarized 40.4% of the variability existing in the abundance data of the macroinvertebrate communities and explained 62.8% of their relationship with the environmental variables ( Table 2 ). The first redundancy analysis axis showed stronger positive correlation with urbanization and land use and a negative correlation with altitude ( Table 3 , Figure 4 ). The second axis showed a stronger negative correlation with EC ( Table 3 , Figure 4 ). In general, the first axis segregated samples from 3 ^rd^ and 4 ^th^ order segments of the VMR and TRR from samples from the IMR microbasin and 1 ^st^ and 2 ^nd^ order segments of the VMR and TRR ( Figure 4 ). This segregation was determined especially by urbanization, land use, and RD ( Figure 4 ).

**Table 2.**
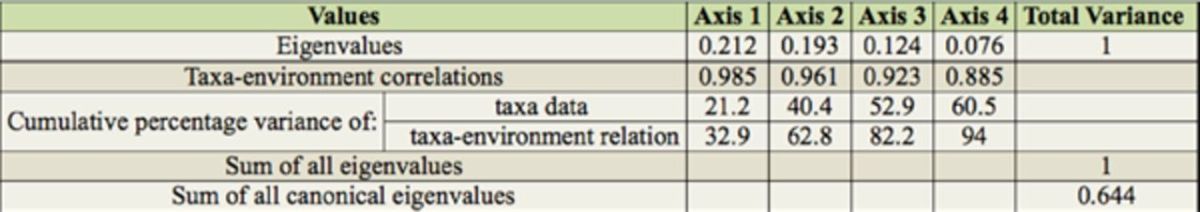
Eigenvalues, taxa-environment correlation coefficients, and cumulative percentage explained by the first four redundancy analysis axes for benthic macroinvertebrate communities of the Vacacaí-Mirim (VMR) (August 2008), Ibicuí-Mirim (IMR), and Tororaipí (TRR) (August 2009) river microbasins and environmental variables, Rio Grande do Sul, Brazil.

**Table 3.**
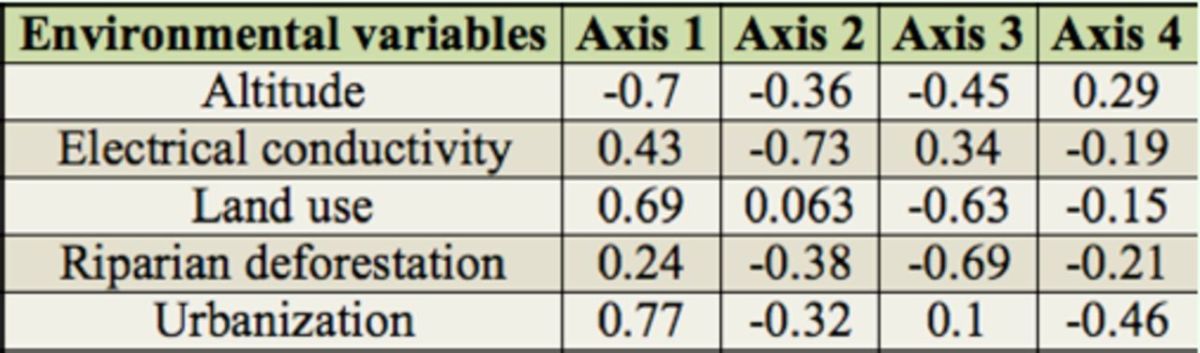
Redundancy analysis inter-set correlations among the first axes and the environmental variables of the Vacacaí-Mirim (August 2008), Ibicuí-Mirim, and Tororaipí (August 2009) rivers, Rio Grande do Sul, Brazil.

However, the second axis segregated two groups: 1) samples of the 3 ^rd^ order of IMR and TRR microbasins, and 2) samples of the VMR and 1 ^st^ , 2 ^nd^ and 4 ^th^ orders of the IMR microbasin ( Figure 4 ). This segregation was established by the higher EC, RD, and altitude recorded in group 2. Additionally, segregation was detected among samples from 1 ^st^ and 2 ^nd^ order segments and samples from 3 ^rd^ and 4 ^th^ order segments of the VMR, which was related to higher EC, RD, and urbanization recorded in 4 ^th^ order ( Figure 4 ). On the second axis, segregation also occurred between the sample from the 4 ^th^ order segment of the TRR, the other samples from this river, and the samples from the IMR. The clustering of these samples was related to the lower EC found in the samples of the cluster, while the segregation of the 4 ^th^ order segment of the TRR was related to the muddy substrate (finer granulometry) that was present only at this site ( Figure 4 ).

Macroinvertebrate taxa could be classified into five groups based on the environmental variables investigated ( Figure 4 ): i) taxa showing close association with high EC, RD, and urbanization, such as Naididae, Tubificidae (Oligochaeta), Hirudinea, *Dicrotendipes, Rheocricotopus, Thienemanniella, Kiefferulus,* and Gen et sp. indet. D (Chironomidae); ii) taxa present in sites with heavy land use, such as *Ablabesmyia, Chironomus, Larsia, Nanocladius* and *Parachironomus* (Chironomidae); iii) taxa present in lower altitudes, such as *Caladomyia, Pentaneura, Tanypus* (Chironomidae), Ceratopogonidae, *Paracloeodes, Apobaetis, Tricorythodes* (Ephemeroptera), Alluroididae, and Enchytraeidae (Oligochaeta); iv) taxa present in higher altitudes, such as *Camelobaetidius* and *Tricorythopsis* (Ephemeroptera), *Heterelmis, Microcylloepus,* and Psephenidae (Coleoptera), *Smicridea* (Trichoptera), *Lopescladius* (Chironomidae), and Tabanidae; and v) sensible taxa, which were present in sites with low impacts of urbanization and land use, such as *Gripopteryx, Paragripopteryx,* and *Tupiperla* (Plecoptera), *Americabaetis, Caenis,* and *Farrodes* (Ephemeroptera), *Neoelmis* (Coleoptera), *Beardius, Cardiocladius,* and *Parametriocnemus* (Chironomidae), Simuliidae, and *Aegla* (Aeglidae).

The partitioning of the variance summarized in redundancy analysis revealed that 29.1% was explained by purely anthropic variables (land use, RD, and urbanization), 18.8% by purely natural variables (altitude and EC), and 16.4% by intersect of the natural and anthropic variables. 35.4% of the variance remained unexplained.

## Discussion

### Environmental variables

The similarities among the natural environmental factors of the three microbasins studied were determined by their geomorphological context. All the microbasins were located in a region of landscape transition, among the Planalto (uplands) and the Depressão Central (lowlands). Small streams located in higher altitudes have coarser sediment and more highly-oxygenated water. Coarser sediments can promote water oxygenation by creating turbulence ( Maier 1978 ). The VMR showed higher values of pH, EC, and DO than the TRR and IMR microbasins, possibly due to differences in surficial geology. Many headwaters of the VMR originated over the basalts of the Serra Geral Formation ( Ferreira et al. 2009 ), while headwaters of the other microbasins originated over the sandstones of the Botucatu and Caturrita formations ( Rodrigues and Werlang 2011 ). Electrical conductivity can be influenced by geological differences ( Melo 2009 ), although anthropic activities can also determine EC values (e.g., Strieder et al. 2006a ; Allan and Castillo 2007 ).

Landscape similarities among the microbasins resulted in little variation of natural factors along the longitudinal gradient as stream order changed. However, some anthropic factors did not follow this gradient. In general, bank erosion, the consequent artificial input of fine sediments into the stream beds, and urbanization tend to increase downstream, in lowlands where human activities are more numerous ( Vischetti et al. 2008 ). This trend was also observed in the study area. However, land use and RD did not follow this gradient. Agriculture and grazing occurred in the slope of the Planalto in the form of rice and soybean plantations and farming of sheep and cattle ( Samuel-Rosa et al. 2011 ) where natural vegetation cover would otherwise be present. Surprisingly, the best-preserved riparian vegetation was found in the 4 ^th^ order segment o the TTR. Brazilian federal laws for protecting the riparian vegetation, especially along large order segments, prevent riparian deforestation, explaining the lack of a positive correlation between RD and land use in this area. This condition was also observed in other studies ( Tate and Heiny 1995 ; Henriques-Oliveira and Nessimian 2010 ; Aristi et al. 2012 ). However, these laws are not always followed, and RD did not show a gradient.

### Structure of macroinvertebrate communities in the study area

The total richness (129 taxa) registered was relatively high and comparable to richness registered in studies of other streams of the world, in which macroinvertebrates assemblages were investigated at the genus level ( Ortiz and Puig 2007 ; Oliveira and Callisto 2010 ; Miserendino et al. 2011 ; Egler et al. 2012 ). High macroinvertebrate richness has been related to many factors, including environmental heterogeneity ( Beisel et al. 2000 ; Voelz and McArthur 2000 ), coarse granulometry ( Heino 2000 ; Wu and Legg 2007 ), presence of aquatic vegetation ( Souza-Franco et al. 2009 ), middle-order rivers ( Minshall et al. 1985 ; Callisto et al. 2004 ), and also to areas of relief transition ( Statzner and Higler 1986 ; Floss et al. 2012). Additionally, many studies have shown that macroinvertebrate richness is negatively related to human activities, such as logging ( Martel et al. 2007 ), plant crops and pasture ( Jacobsen and Marín 2008 ), domestic-sewage discharge ( Cortezzi et al. 2009 ), agriculture ( Delong and Brusven 1998 ; Roque et al. 2003 ), and land erosion ( Larsen et al. 2009 ). Thus, the richness recorded in this study suggests that the transitional landscape in which the microbasins were located is capable of compensating for the negative influences that anthropic activities can cause in the structure of macroinvertebrate communities.

The high abundance of Simuliidae, Naididae, Chironomidae *(Cricotopus),* and Baetidae *(Americabaetis),* as well as the low abundance and richness of other families of Ephemeroptera, and of taxa of Trichoptera, shows that the microbasins analyzed suffered from anthropic environmental impacts. Naididae are known as tolerant and are related to anthropic activity ( Alves and Lucca 2000 ; Alves et al. 2006 ). Simuliidae is one of the most abundant families of macroinvertebrates in rivers with high current velocity ( Gaona and Andrade 1999 ; Bolaño et al. 2003 ) and organic-matter content ( Vuori and Joensuu 1996 ; Strieder et al. 2006b ). In many cases, increased presence of organic-matter originates from sewage discharge, logging of riparian vegetation, and erosion due to agricultural activities. The family Baetidae is considered somewhat sensitive to biotic indices ( Hilsenhoff 1988 ) and has been found in urban streams ( Moyo and Phiri 2002 ). Some baetid species are frequent in sites contaminated by heavy metals ( Márques et al. 2001 ). *Americabaetis* is one of the most common genera of Baetidae and has plastic environmental requirements, occurring in most habitats ( Domínguez et al. 2006 ). *Cricotopus* has been associated with eroded areas ( Sanseverino and Nessimian 2001 ) with no riparian vegetation ( Siqueira and Trivinho-Strixino 2005 ), urbanization ( Jones and Clark 1987 ; Rae 1989 ), and metal contamination ( Yasuno et al. 1985 ).

The interaction of natural variation and local antropic influence in each microbasin with its longitudinal gradient is reflected in different levels of dominance in taxa and in a large number of exclusive taxa in each microbasin. In general, taxa such as *Chironomus,* Tubificidae, and Naididae dominate in larger streams under the influence of human activities ( Marchese 1987 ; Alves and Lucca 2000 ; Siqueira and Trivinho-Strixino 2005 ), while Elmidae, Tipulidae, Psichodidae, *Cricotopus, Paragripopteryx* and *Gripopteryx* predominate in more well-preserved small order streams ( Brown 1987 ; Froehlich 1999 ). In the study area, anthropic influence did not follow a longitudinal gradient, resulting in a lack of patterning of spatial distribution of the communities between microbasins, as demonstrated by the ANOSIM. Only lower stream order communities were segregated, as shown by the non-metric multidimensional scaling. The higher richness of Ephemeroptera, Plecoptera, and Trichoptera found in low-order segments (see Supplementary Data) suggests that these segments conserved some of the original conditions of the streams, since high richness of these taxa has been related to low-order streams with clean and well oxygenated waters ( Rosenberg and Resh 1993 ; Crisci-Bispo et al. 2007 ; Dinakaran and Anbalagan 2007 ). Therefore, in spite of the high abundance of tolerant organisms in all the stream segments, the presence of EPT in low-order sites suggests that these segments had better and more natural environmental conditions than those of the larger segments.

Richness registered in each microbasin and stream order reflected variations in natural and anthropic factors more adequately than the assessment of dominant or exclusive taxa. For example, the 3 ^rd^ and 4 ^th^ order segments showed higher richness than 1 ^st^ and 2 ^nd^ order segments, as observed previously ( Minshall et al. 1985 ; Callisto et al. 2004 ). However, if segments were analyzed separately, the highest richness was found in segments with a medium degree of impact, such as the 2 ^nd^ order o the VMR, 4 ^th^ order of the IMR, and 1 ^st^ and 4 ^th^ order segments of the TRR (see Supplementary Data). Hence, the highest richness recorded in these segments could be explained by the intermediate disturbance hypothesis ( Connell 1978 ), which suggests that moderately impacted sites allow the coexistence of the highest numbers of both sensitive and tolerant species ( Townsend et al. 1997 ; Roxburgh et al. 2004 ).

### Influence of natural and anthropic variables on spatial distribution of communities

In the study area, anthropic factors were shown to be as important as natural factors in explaining the spatial distribution of communities and their taxa. The degree of urbanization and land use tended to change along the longitudinal gradient of the streams studied, which is ultimately a result of the change in altitude. The redundancy analysis shows that as altitude decreased, the level of urbanization and land use increased As previously discussed, the level of urbanization and land use are higher in larger-order streams. Riparian deforestation is also important in explaining our results but it does not correlate with altitude. RD can determine changes in macroinvertebrate communities related to composition, abundance, and richness ( Lenat and Crawford 1994 ; Miserendino et al. 2011 ). Many studies have demonstrated that riparian vegetation provides organic matter and nutrients necessary for high richness of macroinvertebrates in aquatic ecosystems (e.g., Bunn et al. 1999 ; Cummins 1974 ; Cummins et al. 1989 ; Rios and Bailey 2006 ;). Impoverished and homogeneous macroinvertebrate communities are found in streams with less riparian vegetation ( Kay et al. 2001 ; Corbi and Trivinho-Strixino 2008 ).

EC also plays an important role in explaining the distribution of macroinvertebrate taxa. It has been considered one of the most important environmental variables affecting the community structure of benthic macroinvertebrates ( Allan and Castillo 2007 ; Melo 2009 ). Differences in the geology of the drainage areas of the microbasins could explain the higher EC values throughout the VMR sites, since many of its headwaters arose in and flowed over basalts, while the headwaters of the other rivers flowed over sandstone. Rainfall can also affect EC values because high precipitation carries ions into the river basins, increasing their EC ( Esteves 1998 ). However, precipitation in the month prior to the sampling in the TRR and IMR microbasins was higher (164.5 mm) than in the VMR basin (99.8 mm). Therefore, the EC showed a contrary pattern to what would be expected, which reinforces the hypothesis of a geological origin for differences in the EC values among microbasins. Levels of EC did not show a longitudinal gradient.

Interactions between natural and anthropic factors also explain the results obtained in the present study. In general, high EC values are related to sites impacted by urbanization, i.e, untreated industrial and domestic sewage, extensive agriculture, or both ( Cuffney et al. 2000 ; Strieder et al. 2006a ; Allan and Castillo 2007 ). Some of the VMR tributaries, mainly in the lowlands, drained the urban area of Santa Maria Municipality, consequently receiving domestic-sewage discharge. These areas were surrounded by intensive agricultural land use along the river’s banks. Thus, higher EC values may reflect the manifold impacts of geological origins and of an urban area, since EC values were higher in the segments downstream of the city (3 ^rd^ and 4 ^th^ orders).

In this context, anthropic and natural factors, as well as interactions between these factors, influenced some macroinvertebrate taxa. High values of EC, RD, and urbanization are related to the occurrence of Naididae, Tubificidae (Oligochaeta), Hirudinea, *Dicrotendipes, Rheocricotopus, Thienemanniella, Kiefferulus* and Gen et sp. indet. D (Chironomidae). Naididae are found in many aquatic habitats, showing especially high abundance at sites with stony substrates and rich organic content ( Alves and Lucca 2000 ). Tubificidae are found in sediments with abundant organic matter ( Montanholi-Martins and Takeda 1998 ). They also possess respiratory pigments, which improve respiration in environments with low levels of DO ( Miserendino 1995 ). *Dicrotendipes* are typical of calm water with low current velocity, inhabit fine sediment, and are resistant to certain types of environmental degradation, such as the absence of riparian forest ( Pinder and Reiss 1983 ; Epler 2001 ). High land use is correlated with *Ablabesmyia, Chironomus, Larsia, Nanocladius,* and *Parachironomus* (Chironomidae). *Ablabesmyia* are typical of lentic environments and sandy substrates. Together with *Chironomus* and *Larsia, Ablabesmyia* are also resistant to environmental degradation, such as the absence of ripari an forest ( Pinder and Reiss 1983 ; Epler 2001 ). In lower altitudes, *Caladomyia, Paracladius, Pentaneura, Tanypus, Tanytarsus* (Chironomidae), Ceratopogonidae, *Apobaetis, Tricorythodes* (Ephemeroptera), and Enchytraeidae (Oligochaeta) were more abundant. *Caladomyia* is characteristic of lentic environments associated with litter, higher temperatures, and the presence of macrophytes ( Epler 2001 ). *Tanypus* and *Paracladius* have also been found in lentic environments with fine sediments and low current velocity ( Pinder and Reiss 1983 ; Epler 2001 ). Hepp et al. (2013) discovered an association of *Apobaetis* with higher pH values and lower DO levels. In higher altitudes, *Camelobaetidius* and *Tricorythopsis* (Ephemeroptera), *Heterelmis, Microcylloepus* and Psephenidae (Coleoptera), *Smicridea* (Trichoptera), *Lopescladius* (Chironomidae) and Tabanidae were common. *Camelobaetidius* is sensitive to pollution ( Buss and Salles 2007 ). *Heterelmis, Microcylloepus,* and *Smicridea* are reportedly related to gravelly substrates, well-oxygenated waters, and environmental integrity ( Spies et al. 2006 ; Passos et al. 2007 ). Sensible taxa were found in sites with low impacts of urbanization and land use. Sensible taxa, defined as those present in sites with low impacts of urbanization and land use, were *Gripopteryx, Paragripopteryx* and *Tupiperla* (Plecoptera), *Americabaetis, Caenis* and *Farrodes* (Ephemeroptera), *Neoelmis* (Coleoptera), *Beardius, Cardiocladius* and *Parametriocnemus* (Chironomidae), Simuliidae and *Aegla* (Aeglidae). *Gripopteryx* and *Paragripopteryx* are sensitive to pollution, being associated with low human disturbance and high water flow ( Roque et al. 2003 ; Hepp et al. 2013 ). *Tupiperla* and *Caenis* are found in colder and well-oxygenated waters ( Hepp et al. 2013 ). Simuliidae have been related to environmental integrity because they also need well-oxygenated waters ( Strieder et al. 2006b ). Thus, various taxa identified in the VMR, IMR, and TRR support previous findings regarding preferences for certain habitats and their environmental quality.

### Final Remarks

The macroinvertebrate communities of the three microbasins showed high diversity, with a predominance of tolerant taxa. The structure of the communities showed differences among the microbasins, revealing the importance of landscape factors. These differences were influenced by differences in geology and land use in the microbasins, which affected abiotic factors such as EC and substrate granulometry. The longitudinal gradient played an important role, as predicted by the river continuum concept. However, local anthropic changes, which were not perfectly correlated with the longitudinal gradient, also affected the composition and richness of the communities, showing the importance of the intermediate disturbance hypothesis in explaining the structure of the macroinvertebrate communities.

Future inventories of riverine macroinvertebrate communities and their spatial distributions must be carried out using sampling designs that account for differences in stream order, microbasins, and basins’ locations in order to obtain data that faithfully reflect regional diversity. This consideration is especially important with respect to landscape environmental gradients, such as those of the transition from the Planalto to the Depressão Central in southern Brazil, in order to account for fauna associated with different geomorphological compartments. Additionally, it is necessary to document the macroinvertebrate communities along environmental gradients of land uses and anthropic impacts in order to suggest appropriate strategies for conserving environmental integrity. The need for such documentation is illustrated by the distinct differences in macroinvetebrate community structures observed in the stream segments evaluated in the present study.

## Abbreviations

DO: dissolved oxygen
EC: electrical conductivity
IMR: Ibicuí-Mirim River
RD: riparian deforestation
TRR: Tororaipí River
VMR: Vacacaí-Mirim River
